# Influenza vaccine effectiveness against outpatient acute respiratory illness with laboratory-confirmed influenza, United States, 2024–25 season

**DOI:** 10.64898/2026.03.24.26348229

**Published:** 2026-03-26

**Authors:** Jessie R. Chung, Ashley M. Price, Stacey L. House, Jamie Mills, Karen J. Wernli, Magali Sanchez, Emily T. Martin, Ivana A. Vaughn, Vel Murugan, Joanna Kramer, Elie A. Saade, Kiran Faryar, Manjusha Gaglani, Chandni Raiyani, Richard K. Zimmerman, Louise H. Taylor, Olivia L. Williams, Emmanuel B. Walter, Juliana DaSilva, Marie K. Kirby, Min Z. Levine, Rebecca Kondor, Emma K. Noble, Kelsey M. Sumner, Sascha R. Ellington, Brendan Flannery, US Flu VE Network Investigators

**Affiliations:** 1 Influenza Division, US Centers for Disease Control and Prevention, Atlanta, GA, USA; 2 Washington University School of Medicine in St. Louis, Department of Emergency Medicine, St. Louis, MO, USA; 3 Kaiser Permanente Washington Health Research Institute, Seattle, WA, USA; 4 Kaiser Permanente Bernard J. Tyson School of Medicine, Pasadena, CA, USA; 5 University of Washington, Department of Epidemiology, Seattle, WA, USA; 6 University of Michigan School of Public Health, Ann Arbor, MI, USA; 7 Henry Ford Health, Department of Public Health Sciences, Detroit, MI, USA; 8 Biodesign Center for Personalized Diagnostics, College of Health Solutions and Health Observatory, Arizona State University, Tempe, AZ; 9 Phoenix Children’s Hospital, Phoenix, AZ; 10 University Hospitals Cleveland Medical Center, Case Western Reserve University School of Medicine, Cleveland, OH; 11 Baylor Scott & White Health, Temple, TX, USA; 12 Baylor College of Medicine, Temple, TX, USA; 13 University of Pittsburgh School of Medicine, Department of Family Medicine, Pittsburgh, PA, USA; 14 Duke Human Vaccine Institute, Duke University School of Medicine, Durham, NC, USA; 15 Duke University School of Medicine, Department of Pediatrics, Durham, NC

**Keywords:** Influenza, vaccine effectiveness, vaccination

## Abstract

**Background:**

Influenza A(H1N1)pdm09 and A(H3N2) viruses predominated during the 2024–25 U.S. influenza season. We estimated influenza vaccine effectiveness (VE) in the United States against mild-to-moderate outpatient influenza illness by influenza type and subtype in the 2024–25 season.

**Methods:**

We enrolled outpatients aged ≥8 months with acute respiratory illness symptoms including cough in 7 states. Upper respiratory specimens were tested for influenza type/subtype by reverse-transcriptase polymerase chain reaction (RT-PCR). Influenza VE was estimated with a test-negative design comparing odds of testing positive for influenza among vaccinated versus unvaccinated participants controlling for age, study site, underlying health status, and month of illness onset. We also estimated VE of current season vaccination among adults stratified by prior season vaccination status.

**Results:**

Among 6,793 enrolled patients, 2,016 (30%) tested positive for influenza including 961 A(H3N2), 770 A(H1N1)pdm09, and 183 B/Victoria. Overall vaccine effectiveness against any influenza illness was 33% (95% Confidence Interval [CI]: 24 to 41): 27% (95% CI: 14 to 39) against influenza A(H3N2), 37% (95% CI: 24 to 48) against A(H1N1)pdm09, and 40% (95% CI: 12 to 59) against B/Victoria. VE did not differ based on whether or not participants had received influenza vaccine the previous season.

**Conclusions:**

Influenza vaccination during the 2024–25 season protected against circulating influenza viruses, reducing the risk of outpatient medically attended influenza overall by approximately one-third among people who were vaccinated.

## Introduction

Influenza causes annual epidemics of acute respiratory illness. During the 2024–25 season, influenza caused an estimated 43 million – 76 million illness episodes, 19 million – 34 million medical visits, 560,000 – 1.2 million hospitalizations and 38,000 – 110,000 influenza-associated deaths in the United States^1^. The 2024–25 influenza season was the first since 2017–18 to be classified as high severity for all age groups^2,3^. For prevention of influenza and complications of influenza virus infection, annual influenza vaccination has been recommended in the United States for all persons aged ≥6 months who do not have contraindications since 2010^4^. Effectiveness of influenza vaccination is monitored to assess protection against circulating influenza viruses and inform annual influenza vaccine updates. Influenza vaccine effectiveness (VE) against medically attended outpatient influenza in the U.S. has ranged from 19% to 60% since 2009^5^. Since 2013, U.S. Centers for Disease Control and Prevention (CDC) has routinely published interim and end-of-season estimates of influenza VE. Annual estimates of the burden averted from influenza vaccination were introduced in 2010. From 2010–11 to 2023–24, influenza vaccination is estimated to have prevented 35 million influenza-associated healthcare visits, 1 million hospitalizations, and 85,000 deaths in the United States ^6^.

In February 2025, interim estimates from four U.S. VE platforms showed significant protection against outpatient and inpatient influenza illness^7^. Here we report updated VE for the 2024–25 season against outpatient medically attended influenza overall by influenza type and A subtype, age group, time since vaccination, and prior season vaccination status.

## Methods

### Study population

The US Flu VE Network has been described previously ^8^. Briefly, this public health surveillance network enrolls participants aged 8 months or older with a mild to moderate severity acute respiratory illness (ARI) ≤7 days duration including new or worsening cough. Patients were enrolled at participating outpatient healthcare facilities, urgent care and emergency departments at sites in Arizona, Michigan, Missouri, Ohio, Pennsylvania, Texas, and Washington. Duke University served as the data coordinating institution for the network. Enrollment ramped up or down at each site based on local evidence of increasing or decreasing influenza activity, respectively (Supplemental Table 1). Trained staff interviewed participants or their parent/guardian to obtain demographic and clinical information and inquired about receipt of current season influenza vaccination. Underlying chronic health conditions were defined as either self-report or electronic medical record documentation within the previous year of medical encounters with International Classification of Diseases (ICD-10) codes for high-risk conditions associated with influenza-related complications^9^. The study protocol was reviewed by CDC, determined to be public health surveillance, and conducted consistent with applicable federal law and CDC policy (45 CFR 46.102(l)(2)). Participants or their parent/guardian provided written or oral consent.

### Influenza vaccination

Northern Hemisphere 2024–25 influenza vaccines were trivalent and included two influenza A antigens and one influenza B antigen: A(H3N2) clade 2a.3a.1 subclade J (egg-based A/Thailand/8/2022 or cell-based A/Massachusetts/18/2022), A(H1N1)pdm09 clade 5A.2a.1 subclade C.1.1 (egg-based A/Victoria/4897/2022 or cell-based A/Wisconsin/67/2022) and B/Victoria clade V1A.3a.2 subclade C (egg- or cell-based B/Austria/1359417/2021)^4^. The recommended A(H3N2) vaccine viruses were updated in February 2024; A(H1N1)pdm09 and B/Victoria vaccine viruses were the same as 2023–24 (Supplemental Table 2). Sources of influenza vaccine documentation included electronic health records (EHR), state immunization information systems, and vaccine provider information. For patients or parent/guardian reporting children’s vaccination ≥14 days before illness onset, study staff determined plausibility based on reported date and location of vaccination if self-reported vaccination was not captured in electronic immunization records; patients with one or more documented doses of any licensed influenza vaccine product after July 1, 2024 or plausible self-report of influenza vaccination without documentation were considered vaccinated. Influenza vaccine type and prior season (i.e., 2023–24) influenza vaccination status were obtained from electronic immunization records only.

### Laboratory methods

At enrollment, study staff collected nasal and oropharyngeal swabs (nasal only for children younger than 2 years of age) for respiratory virus testing. Respiratory specimens were tested for influenza (including type and influenza A subtype) and SARS-CoV-2 viruses by reverse-transcriptase polymerase chain reaction (RT-PCR) at site laboratories. Case-patients were classified based on study or clinical influenza RT-PCR test result; control-patients were those who tested negative for influenza virus and SARS-CoV-2 infection. Influenza-positive specimens with RT-PCR cycle threshold values <31 were genetically characterized using whole genome sequencing (WGS) at CDC with each sample’s hemagglutinin (HA) clade/subclade assigned using NextClade and based on its consensus sequence^10^.

Serum samples were obtained at enrollment from a subset of patients who agreed to venipuncture. Serum was separated and stored at −20°C or colder at study sites until shipment to CDC for serologic analysis. The microneutralization assay^11^ (MN) was used to quantify antibody levels of A(H3N2)-positive participants at the acute timepoint against the predominant circulating A(H3N2) virus in the Flu VE Network during 2024–25 (A/Wisconsin/154/2024, HA subclade J.2). Assays were conducted as previously described^12^ (Supplemental Methods). Control sera from uninfected, influenza-negative participants were matched to case-patient sera based on participant age and month of illness onset. Antibody levels are reported as geometric mean titers (GMT) and 95% confidence intervals. GMTs of those vaccinated and unvaccinated in the prior season were compared using a linear regression model controlling for influenza case status, stratified by current season vaccination status. P-values <0.05 were considered statistically significant.

### Statistical analysis

Influenza VE was estimated with a test-negative design comparing odds of testing positive for influenza among vaccinated versus unvaccinated participants^13^. VE is calculated as (1 – aOR) × 100 expressed as a percent, where aOR is the adjusted odds ratio for RT-PCR-confirmed influenza from multivariable logistic regression models. Models were adjusted a priori for study site, participant age (as age group for all-ages estimates and year of age for age group-specific estimates) at enrollment, presence of ≥1 underlying health conditions (either from documentation or participant/parent/guardian report), and month of illness onset. Additional covariates (sex, race and ethnicity, self-rated general health status, and interval between illness onset and enrollment) were considered for inclusion using a threshold for inclusion of ≥5% change in the aOR. Statistical significance was defined as 95% confidence intervals that did not include zero. We estimated VE overall by influenza A and B type, influenza subtype, and age group (8 months – 4 years, 5–17 years, 8 months – 17 years, 18–49 years, 50–64 years, and ≥65 years). Age group-specific estimates were combined when models did not converge. We estimated VE by age group against any influenza illness by time since vaccination using intervals of 14–60 days, 60–120 days, and >120 days since vaccination compared with unvaccinated participants.

Taking a patient-centered approach^14,15^, we evaluated the added benefit of current season vaccination among patients aged ≥18 years who were vaccinated or unvaccinated the preceding season (2023–24). We used an interaction term of current and prior season vaccination to compare VE estimates^16^. We restricted these analyses to four study sites (MI, PA, TX, and WA) where the majority of that season’s influenza vaccine doses were documented in electronic sources.

## Results

### Participant characteristics

From October 6, 2024–May 10, 2025, 9,782 patients with acute respiratory illness were enrolled at time of presentation for ambulatory care. A total of 2,989 (31%) patients did not meet inclusion criteria for primary VE analyses. Most of the excluded patients already had a clinical influenza diagnostic test result before enrollment which could potentially bias the reported clinical and vaccination information (46% of exclusions). Patients enrolled outside periods of influenza circulation (24% of exclusions) and those who tested RT-PCR positive for SARS-CoV-2 infection (17% of exclusions) also were excluded because influenza and COVID-19 vaccination have been highly correlated^17^ (Supplemental Figure 1). Among 6,793 patients who met inclusion criteria, 2,016 (30%) tested positive for influenza virus infection (Table 1): 1,830 (91%) influenza A (961 [53%] A(H3N2), 770 [42%] A(H1N1)pdm09, 99 [5%] undetermined subtype), and 183 (9%) for influenza B; 3 (<1%) had influenza A(H1N1)pdm09 and A(H3N2) virus coinfections. Weekly number of influenza-positive participants peaked in week 5 of 2025 (Supplemental Figure 2). A total of 2,262 (33%) participants were considered vaccinated against influenza for the 2024–25 season. Of these, 323 (14%) had plausible self-reported vaccination without documentation in electronic immunization record.

Influenza-positive case patients were more likely to report better general health and sought care earlier compared to test-negative control patients; case patients were less likely to have an underlying health condition than controls (Table 1). The proportion of participants vaccinated differed by study site, sex, age group, race and ethnicity, general health status, presence of an underlying health condition, and number of days between illness onset to enrollment (Table 1). Most participants vaccinated in 2024–25 (1,318, 68%) were also vaccinated in the 2023–24 season. Among participants aged <65 years, the type of influenza vaccine received was documented in electronic immunization records for 1,295 (75%) of 1,732 vaccinated patients: 815 (63%) received egg-based inactivated influenza vaccine (807 [99%] standard dose [IIV3-SD], 5 [1%] live-attenuated influenza vaccine, 2 [<1%] high-dose [IIV3-HD], and1 [<1%] adjuvanted [aIIV3]), and 480 (37%) received non-egg-based vaccines (424 [88%] cell culture-based IIV3 [ccIIV3] and 56 [12%] recombinant influenza vaccine [RIV3]). Among adults aged ≥65 years at enrollment, influenza vaccine type was documented for 464 (88%) of 530 vaccinated participants: 439 (95%) received a preferentially recommended vaccine^4^ (235 [54%] IIV3-HD, 179 [41%] aIIV3, and 25 [6%] RIV3).

### Genetic characterization

Of 1,478 influenza-positive respiratory specimens that met criteria for WGS, 1,380 (93%) viruses were successfully sequenced: 692 A(H3N2), 571 A(H1N1)pdm09 and 117 B/Victoria (Supplemental Table 3). A(H1N1)pdm09 viruses belonged to two HA clades: 406 (71%) to 5a.2a.1 and 165 (29%) to 5a.2a. All A(H3N2) viruses belonged to the 2a.3a.1 HA clade; most (479, 69%) belonged to subclade J.2, and smaller proportions belonged to J.2.2 (111, 16%), J.2.5 (77, 11%), J.2.1 (6, 1%), J.2.4 (8, 1%), or J.1.1 (1, <1%). All influenza B/Victoria viruses belonged 3a.2 HA clade C: 43% subclade C.3.1, 27% C.5.1, 11% C.5.6, 9% C.5.7, 4% C.5.6.1, 3% C.5, and 2% C.3.2.

### Overall influenza vaccine effectiveness

For the period from October 2024 through May 2025, the overall VE against medically attended outpatient influenza A and B viruses was 33% (95% confidence interval [CI]: 24 to 41); VE was not statistically significant among patients aged ≥65 years (VE: 4%, 95%CI: −42 to 35) (Table 2). VE was 27% (95%CI: 14 to 39) against A(H3N2), 37% (95%CI: 24 to 48) against influenza A(H1N1)pdm09, and 40% (95%CI: 12 to 59) against influenza B/Victoria. VE against influenza A(H3N2) and A(H1N1)pdm09 was highest among children aged 8 months to 4 years (41% and 73%, respectively).

### Effects of time since influenza vaccination

The median time between influenza vaccination and illness onset was 108 days (interquartile range [IQR] 65–146 days). Overall and for each age group, VE point estimates were highest among participants vaccinated 14–59 days prior to illness onset compared to those with longer intervals although confidence intervals overlapped (Supplemental Table 4).

### Effects of prior season influenza vaccination

Among 2,637 participants from the four sites included in the analysis, 817 (31%) individuals were vaccinated against influenza in both the current (2024–25) and prior (2023–24) seasons, 442 (17%) were vaccinated in the current season only, 224 (8%) were vaccinated in the prior season only, and 1,154 (44%) were unvaccinated in both seasons (Table 3). Participants unvaccinated in both seasons were more likely to test positive compared to other groups. Among participants who were not vaccinated in the prior season, VE of current season vaccination against any influenza was 33% (95% CI: 13 to 49) compared with 17% (95%CI: −20 to 43) among participants who were vaccinated in both seasons (p-value for interaction = 0.26). VE against A(H3N2) or A(H1N1)pdm09 did not differ by prior season vaccination status (p-values for interaction 0.81 and 0.32, respectively). Sample sizes were insufficient to estimate VE against influenza B/Victoria by prior vaccination status.

### Serum antibody levels to influenza A(H3N2)

Sera from 232 adults (102 A(H3N2)-positive participants and 130 influenza-negative participants) were tested by the microneutralization assay (Supplemental Table 5). Acute-phase antibody titers against the predominant circulating A(H3N2) virus from HA subclade J.2, A/Wisconsin/154/2024, were higher among test-negative control participants (GMT: 19, 95%CI: 15 to 24) than A(H3N2)-positive cases (GMT: 10, 95%CI: 8 to 12). Among test-negative controls, participants who were vaccinated in the current season had higher antibody levels (GMT: 44, 95%CI: 28 to 68) compared to unvaccinated participants (GMT: 14, 95%CI: 11 to 18). Stratifying by current season vaccination status and controlling for influenza case status, GMT was not associated with prior season vaccination status (p=0.40 for those vaccinated in the current season and p=0.74 for those who did not receive the current season vaccination).

### Sensitivity analyses

VE against any influenza using self- or parent/guardian-reported influenza vaccination only was 23% (95%CI: 11 to 33); VE using EHR-documented data only was 37% (95%CI: 28 to 45) (Supplemental Table 6). Inclusion of participants who could have known their clinical influenza test result at the time of enrollment reduced VE to 28% (95%CI: 19 to 35). All other sensitivity analyses varying inclusion criteria for analysis were all within 6 percentage points of the overall VE reported in the primary analysis.

## Discussion

During the 2024–25 influenza season in the U.S., with co-circulation of influenza A(H3N2) and A(H1N1)pdm09 viruses, influenza vaccination reduced the risk of outpatient medically attended influenza by approximately one-third. Influenza vaccination was associated with lower odds of A(H3N2)-, A(H1N1)pdm09-, and B/Victoria-associated illness with variable levels of protection observed by participant age category. VE may have been affected by genetic and antigenic differences between influenza vaccine components and circulating influenza viruses. Statistically significant protection against medically attended influenza was observed up to 4 months after vaccination with 2024–25 vaccine. VE did not differ by prior season influenza vaccination status, which was supported by similar serum antibody levels against the predominant circulating A(H3N2) virus in participants who were and were not vaccinated against influenza in the prior season. Genetic characterization of influenza viruses and measurement of antibody levels contributed to understanding of protection provided by influenza vaccination in this season.

Seasonal influenza vaccination has been associated with higher protection against A(H1N1)-associated illness compared to protection against A(H3N2). In meta-analyses of VE studies against medically attended outpatient illness, pooled VE against A(H3N2) among all persons ranged from 22–33%, and VE against A(H1N1)pdm09 ranged from 56–61%^18,19^. During 2024–25, influenza vaccination provided protection against A(H1N1)pdm09- and A(H3N2)-associated outpatient visits, with similar estimates of overall VE. Estimated VE against A(H3N2) fell within the range of recent US Flu VE Network estimates during A(H3N2)-predominant seasons. However, VE against A(H1N1)pdm09 was lower than has been seen during seasons when circulating A(H1N1)pdm09 viruses were antigenically similar to vaccine components. During the 2019–20 season, the US Flu VE Network reported decreased protection against an antigenically drifted A(H1N1)pdm09 virus^20^. Observed protection in this Network has been <50% in the subsequent seasons with A(H1N1)pdm09 circulation.

In the 2024–25 season, children in the youngest age group had the highest VE point estimates against influenza A, which is consistent with prior analyses and other studies^21,22^. Among adults aged ≥65 years, who are at higher risk of influenza-associated complications, multiple studies demonstrated protection against influenza A in the 2024–25 season^7^. In this analysis, VE against influenza A was not statistically significant. VE against A(H3N2) in this age group has been consistently low; in meta-analyses, pooled VE against A(H3N2) was 11–24% among older adults^18,19^. The A(H3N2) vaccine components were updated for the 2025–26 season^23^ (Supplemental Table 2). Estimated VE against A(H1N1)pdm09 was lower among older adults compared to pooled meta-analyses estimates (47–62%)^18,19^ but was overlapping with the estimate from the US Flu VE Network from the 2023–24 season (39%, 95%CI: 2 to 62)^8^. In the United States, uptake of adjuvanted and higher-dose influenza vaccines has increased since these vaccines were preferentially recommended for adults aged ≥65 years. However, during the current season when most adults aged ≥65 years received adjuvanted or higher dose vaccines, VE was comparable to prior seasons, despite evidence of improved immunogenicity and effectiveness in some studies^24–26^. Waning immunity might be a contributing factor because individuals aged ≥65 years may be more likely to seek vaccination early^27^. In our study, fewer adults aged ≥65 years were vaccinated within 60 days of illness onset compared to adults aged 18–64 years. Influenza B detections were more numerous among children than adults, and observed VE was lower among children than has been found in prior seasons. Our study included a larger proportion of viruses belonging to the emerging B/Victoria HA subclade C.3.1 (43%) compared to national surveillance data for the season (12.9% of sequenced B/Victoria viruses)^3^, which may have contributed to lower VE against B/Victoria.

We did not detect differences in VE by prior season influenza vaccination status overall or against A(H3N2) or A(H1N1)pdm09 among adults from four study sites with the most complete electronic vaccination records. Higher VE estimates among people vaccinated against influenza only in the current season compared to repeat vaccinees (i.e., those vaccinated in both current and prior seasons) have been reported in several studies^28–30^. In our study, repeat vaccination effects were not statistically significant. Serum antibody titers against the predominantly circulating A(H3N2) virus were consistent with similar protection against laboratory-confirmed influenza gained by current season vaccination regardless of prior season vaccination status. Analyses from the patient perspective over multiple seasons have shown more consistent evidence of the added benefit of current season influenza vaccination even among people vaccinated during the preceding season regardless of antigenic similarities between vaccine components and circulating viruses over two seasons^14,15^.

Findings are subject to several limitations. First, current and prior season influenza vaccination may not have been documented leading to misclassification of vaccinated patients as unvaccinated and underestimating VE. However, documentation was available for 84% of patients with plausible self-report of current season vaccination. Some patients vaccinated against influenza in the prior season may have been inadvertently included in the unvaccinated group in analyses comparing the effect of prior season vaccination. Analyses of prior season vaccine effects were restricted to study sites with high proportions of documented vaccinations. Second, while the test-negative design reduces potential bias due to differences in healthcare seeking, unmeasured confounding related to study enrollment may have affected VE estimates. Findings were robust to changes in inclusion criteria and definitions of exposure status. Patients were systematically tested by highly sensitive and specific RT-PCR for influenza and SARS-CoV-2, reducing misclassification of case status. Enrolled patients were not tested systematically for Respiratory Syncytial Virus (RSV) infection. Missing data on RSV infection were unlikely to have affected influenza VE estimates. As RSV vaccine uptake increases among populations in which vaccination is recommended, systematic testing for RSV in the future may be considered to remove potential bias^31,32^. Third, we did not compare full^4^ versus partial influenza vaccination among children aged <9 years. Receipt of the recommended number of doses has been associated with higher VE among young children compared to partial vaccination^33^. Finally, we did not have serologic testing results for A(H1N1)pdm09- or influenza B-positive cases due to resource constraints.

The results presented in this study demonstrate an overall benefit of receiving the 2024–25 influenza vaccine in the United States; vaccination reduced the risk of outpatient medically attended influenza overall by approximately one-third. In seasons such as 2024–25, when the burden of influenza is high, even modestly effective vaccines can still prevent substantial numbers of influenza-associated medical visits, hospitalizations, and deaths^34,35^.

## Supplementary Material

Supplement 1

## Figures and Tables

**Table 1.** Characteristics of participants enrolled in the US Influenza Vaccine Effectiveness Network for the 2024–25 influenza season

		RT-PCR Test result status					Influenza Vaccination status		
	Total	Influenza-positive		Influenza-negative			Vaccinated		
Characteristic	No.	No.	Column %	No.	Column %	p-value	No.	Row %	p-value
*Overall*	6793	2016	30	4777	70		2262	33	
*Study site*						<0.01			<0.01
Arizona	895	296	15	599	13		120	13	
Michigan	456	116	6	340	7		225	49	
Missouri	559	64	3	495	10		150	26	
Ohio	1120	352	17	768	16		225	20	
Pennsylvania	1514	583	29	931	19		602	40	
Texas	1542	406	20	1136	24		589	38	
Washington	707	199	10	508	11		351	50	
*Sex* ^b^						0.18			<0.01
Female	3952	1148	57	2804	59		1402	36	
Male	2819	861	43	1958	41		853	30	
*Age group (years)*						<0.01			<0.01
8 months-4 years	1108	243	12	865	18		327	29	
5–17	1299	448	22	851	18		295	23	
18–49	2668	854	42	1814	38		732	27	
50–64	886	268	13	618	13		378	42	
≥65	832	203	10	629	13		530	64	
*Race and ethnicity* ^c^									
American Indian or Alaska Native	86	22	1	64	1	0.41	27	32	0.71
Asian	380	129	6	251	5	0.06	164	43	<0.01
Black or African American	1862	504	25	1358	28	<0.01	464	25	<0.01
Hispanic or Latino	1057	304	15	753	16	0.49	266	25	<0.01
Middle Eastern or North African	79	32	2	47	1	0.03	20	25	0.13
Native Hawaiian or Pacific Islander	39	13	1	26	1	0.61	13	33	0.99
White	3768	1131	56	2637	55	0.47	1472	39	<0.01
*Self-rated general health status* ^d^						<0.01			<0.01
Excellent	1777	590	29	1187	25		502	28	
Very good	2290	709	35	1581	33		841	37	
Good	1931	552	27	1379	29		660	34	
Fair/Poor	772	160	8	612	13		253	32	
*Underlying chronic health conditions* ^e^						<0.01			<0.01
None	3390	1105	55	2285	48		858	25	
≥1	3403	911	45	2492	52		1404	41	
*Illness onset to enrollment (days)*						<0.01			<0.01
<3	2838	983	49	1855	39		911	32	
3–4	2307	691	34	1616	34		745	32	
5–7	1648	342	17	1306	27		606	37	
*Received 2023–24 influenza vaccine*	1935	495	25	1440	30	<0.01	1318	68	<0.01

b Sex was missing for 22 participants.

c Participants self-identified all options that applied and could have chosen more than one. 42 participants did not select any of the answer choices.

d Self-rated general health status was missing for 23 participants.

e Underlying chronic health conditions included chronic cardiac diseases and circulatory diseases, chronic pulmonary diseases, diabetes mellitus, chronic renal disease, hemoglobinopathies, immunosuppressive disorders, malignancy, metabolic diseases, liver diseases, neurological/musculoskeletal conditions, cerebrovascular disease, morbid obesity, and endocrine disorders.

**Table 2.** Influenza vaccine effectiveness against outpatient influenza-associated illness visits among patients aged ≥8 months enrolled at US Influenza Vaccine Effectiveness Network sites, October 2024 through May 2025.

	Influenza Positive (Cases)		Influenza Negative (Controls)		Vaccine Effectiveness^a^	
	No. vaccinated/Total	% Vaccinated	No. vaccinated/Total	% Vaccinated	VE %	(95% CI)
**All influenza viruses**						
All ages ≥8 months	575/2016	29	1687/4777	35	33	(24 to 41)
8 months-17 years	146/691	21	476/1716	28	41	(25 to 53)
8 months-4 years	51/243	21	276/865	32	50	(29 to 66)
5–17	95/448	21	200/851	24	26	(−1 to 45)
18–49	205/854	24	527/1814	29	31	(15 to 44)
50–64	99/268	37	279/618	45	37	(12 to 55)
≥65	125/203	62	405/629	64	4	(−42 to 35)
**Influenza A(H3N2)**						
All ages ≥8 months	269/964	28	1687/4777	35	27	(14 to 39)
8 months-17 years	69/333	21	476/1716	28	42	(21 to 57)
8 months-4 years	31/131	24	276/865	32	41	(7 to 63)
5–17	38/202	19	200/851	24	36	(1 to 58)
18–49	101/437	23	527/1814	29	24	(1 to 42)
50–64	40/104	38	279/618	45	33	(−9 to 58)
≥65	59/90	66	405/629	64	−29	(−123 to 25)
**Influenza A(H1N1)pdm09**						
All ages ≥8 months	237/773	31	1687/4777	35	37	(24 to 48)
8 months-17 years	46/238	19	476/1716	28	54	(33 to 68)
8 months-4 years	12/83	14	276/865	32	73	(47 to 86)
5–17	34/155	22	200/851	24	30	(−12 to 56)
18–49	81/297	27	527/1814	29	25	(−2 to 45)
50–64	50/136	37	279/618	45	41	(9 to 32)
≥65	60/102	59	405/629	64	18	(−35 to 50)
**Influenza B/Victoria**						
All ages ≥8 months	44/183	24	1687/4777	35	40	(12 to 59)
8 months-17 years	26/96	27	476/1716	28	25	(−27 to 55)
≥18 years	18/87	21	1211/3061	40	63	(34 to 79)

Abbreviations: CI, confidence interval; VE, vaccine effectiveness

a Models adjusted for study site, age, presence of ≥1 underlying health condition, and month of illness onset. 95% confidence intervals that exclude 0% are considered statistically significant.

b Total influenza cases include 99 influenza A-positives of undetermined subtype and 3 influenza A(H1N1)pdm09 and A(H3N2) coinfections. Coinfections are included as cases of both detected viruses in the table.

**Table 3.** Influenza vaccine effectiveness against outpatient influenza-associated illness visits among participants^a^ aged ≥18 years stratified by prior season (2023–24) vaccination status, October 2024 through May 2025.

	Influenza Positive (Cases)		Influenza Negative (Controls)		Vaccine Effectiveness^b^	
	No. Cases/Row total	(Row %)	No. Controls/Row Total	(Row %)	%	(95% CI)
**Any Influenza** ^c^						
*Prior season unvaccinated*						
Current season vaccinated	128/442	29	314/442	71	33	(13 to 49)
Current season unvaccinated	419/1154	36	735/1154	64	REF	
						
*Prior season vaccinated*						
Current season vaccinated	217/817	27	600/817	73	17	(-20 to 43)
Current season unvaccinated	67/224	30	157/224	70	REF	
						
Influenza A(H3N2)^d^						
*Prior season unvaccinated*						
Current season vaccinated	64/378	17	314/378	83	23	(-9 to 45)
Current season unvaccinated	193/928	21	735/928	79	REF	
						
*Prior season vaccinated*						
Current season vaccinated	89/689	13	600/689	87	25	(-21 to 53)
Current season unvaccinated	38/195	19	157/195	81	REF	
						
**Influenza A(H1N1)pdm09** ^e^						
*Prior season unvaccinated*						
Current season vaccinated	56/370	15	314/370	85	32	(3 to 53)
Current season unvaccinated	167/902	19	735/902	81	REF	
						
*Prior season vaccinated*						
Current season vaccinated	102/702	15	600/702	85	6	(-59 to 44)
Current season unvaccinated	24/181	13	157/181	87	REF	

Abbreviations: CI, confidence interval

a Participants enrolled at study sites in Michigan, Pennsylvania, Texas, and Washington.

b Models adjusted for study site, age, presence of ≥1 underlying health condition, and month of illness onset. 95% confidence intervals that exclude 0% are considered statistically significant.

c The p-value for interaction of prior (2023–24) and current (2024–25) season vaccination was 0.26.

d The p-value for interaction of prior (2023–24) and current (2024–25) season vaccination was 0.81.

e The p-value for interaction of prior (2023–24) and current (2024–25) season vaccination was 0.32.

## Key Points:

Influenza vaccine reduced the risk of outpatient acute respiratory illness due to laboratory-confirmed influenza during the 2024–25 season by a third.

## US Flu VE Network Investigators:

Tamair Curley, Jeremy Russell, Ben Rapp, and Joshua Jackson (Washington University School of Medicine in St. Louis); Erika Kiniry, Brianna Wickersham, Rachael Doud, and Matthew Nguyen (Kaiser Permanente Washington Health Research Institute); Arnold S. Monto, Donny Hearn III, and Caroline K. Cheng (University of Michigan); Maria Santana-Garces, Muniza Hossain, and Shane Bole (Henry Ford Health); Mehal Patel (Phoenix Children’s Hospital); Lora Nordstrom (Valleywise Health); Matthew Scotch, Bradley Bobbett (Arizona State University); Alexia El Khoury, Joy Abou Farah, (University Hospitals of Cleveland) Tracy Lemonovich, (MetroHealth), Curtis Donskey (Louis Stokes Cleveland Veterans Affairs Medical Center); Spencer Rose, Michael Smith, Leah Odame-Bamfo, Kempapura Murthy, Mufaddal Mamawala, Amanda McKillop, Nicole Calhoun, Britan Fairall (Baylor Scott & White Health); Mary Patricia Nowalk, GK Balasubramani, Tracey Conti, David A. Figucia, and Alexandra Weissman (University of Pittsburgh); Natalie A. B. Bontrager, Darren J. Morrow, Wes Rountree, Christopher A. Todd, and Cameron R. Wolfe (Duke University); Lisa Keong, Sydney R. Sheffield, Julia C. Frederick, Malania M. Wilson, Ewelina Lyszkowicz, Yujin Jung, Crystal Holiday, Stacie Jefferson, and Philip Shirk (US Centers for Disease Control and Prevention)
