# Supplementary material for: Influenza vaccine effectiveness against outpatient acute respiratory illness with laboratory-confirmed influenza, United States, 2024–25 season
Source: medRxiv. 2026 Mar 26:2026.03.24.26348229. Preprint. [Version 1] doi: 10.64898/2026.03.24.26348229 (PMC13042114; doi:10.64898/2026.03.24.26348229)
Supplement: Supplement 1 [file NIHPP2026.03.24.26348229v1-supplement-1.pdf]

Supplemental Table 1. Enrollment dates included by site

State	Enrollment Institutions	Enrollment start date	Enrollment end date
Arizona	Arizona State University,<br>Valleywise Health,<br>Phoenix Children's Hospital	October 13, 2024	April 28, 2025
Michigan	University of Michigan,<br>Henry Ford Health	December 9, 2024	May 10, 2025
Missouri	Barnes Jewish Memorial Hospital,<br>St. Louis Children's Hospital	October 7, 2024	April 23, 2025
Ohio	University Hospitals at Cleveland,<br>Cleveland Veterans' Health Administration	October 31, 2024	March 26, 2025
Pennsylvania	University of Pittsburgh Medical Center,<br>Children's Hospital of Pittsburgh	October 30, 2024	May 3, 2025
Texas	Baylor Scott & White Health	October 6, 2024	May 10, 2025
Washington	Kaiser Permanente Washington	October 20, 2024	May 10, 2025

medRxiv preprint doi: https://doi.org/10.64898/2026.03.24.26348229; this version posted March 26, 2026. The copyright holder for this preprint (which was not certified by peer review) is the author/funder, who has granted medRxiv a license to display the preprint in perpetuity.
This article is a US Government work. It is not subject to copyright under 17 USC 105 and is also made available for use under a CC0 license.

Supplemental Table 2. US influenza vaccine strains, 2023–24 through 2025–26 seasons.

	**2023–24**[1]	**2024–25**[2]	**2025–26**[3]
**Egg-based vaccines**			
A(H3N2)	A/Darwin/9/2021	A/Thailand/8/2022	A/Croatia/10136RV/2023
A(H1N1)pdm09	A/Victoria/4897/2022	A/Victoria/4897/2022	A/Victoria/4897/2022
B/Victoria	B/Austria/1359417/2021	B/Austria/1359417/2021	B/Austria/1359417/2021
B/Yamagata	B/Phuket/3073/2013	Not applicable	Not applicable
**Cell culture-based and recombinant vaccines**			
A(H3N2)	A/Darwin/6/2021	A/Massachusetts/18/2022	A/District of Columbia/27/2023
A(H1N1)pdm09	A/Wisconsin/67/2022	A/Wisconsin/67/2022	A/Wisconsin/67/2022
B/Victoria	B/Austria/1359417/2021	B/Austria/1359417/2021	B/Austria/1359417/2021
B/Yamagata	B/Phuket/3073/2013	Not applicable	Not applicable

[1] https://www.who.int/news/item/24-02-2023-recommendations-announced-for-influenza-vaccine-composition-for-the-2023-2024-northern-hemisphere-influenza-season

[2] https://www.who.int/news/item/23-02-2024-recommendations-announced-for-influenza-vaccine-composition-for-the-2024-2025-northern-hemisphere-influenza-season

[3] https://www.who.int/publications/m/item/recommended-composition-of-influenza-virus-vaccines-for-use-in-the-2025-2026-nh-influenza-season

medRxiv preprint doi: https://doi.org/10.64898/2026.03.24.26348229; this version posted March 26, 2026. The copyright holder for this preprint (which was not certified by peer review) is the author/funder, who has granted medRxiv a license to display the preprint in perpetuity.
This article is a US Government work. It is not subject to copyright under 17 USC 105 and is also made available for use under a CC0 license.

Supplemental Figure 1. Exclusion criteria figure

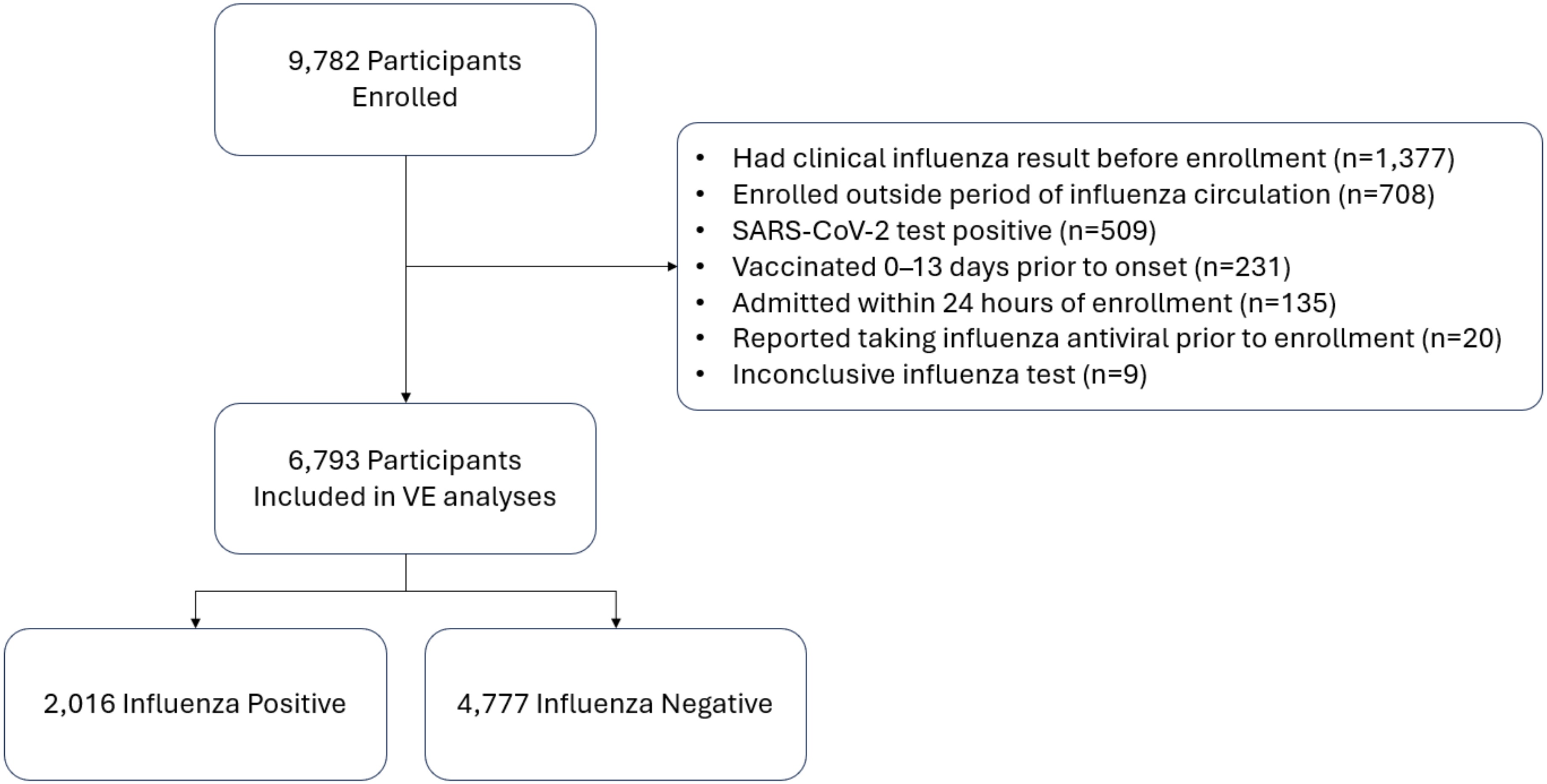

medRxiv preprint doi: https://doi.org/10.64898/2026.03.24.26348229; this version posted March 26, 2026. The copyright holder for this preprint (which was not certified by peer review) is the author/funder, who has granted medRxiv a license to display the preprint in perpetuity. This article is a US Government work. It is not subject to copyright under 17 USC 105 and is also made available for use under a CC0 license.

Supplemental Figure 2. Influenza-positive cases by type/subtype and percent influenza positive by week of enrollment, October 2024 – May 2025.

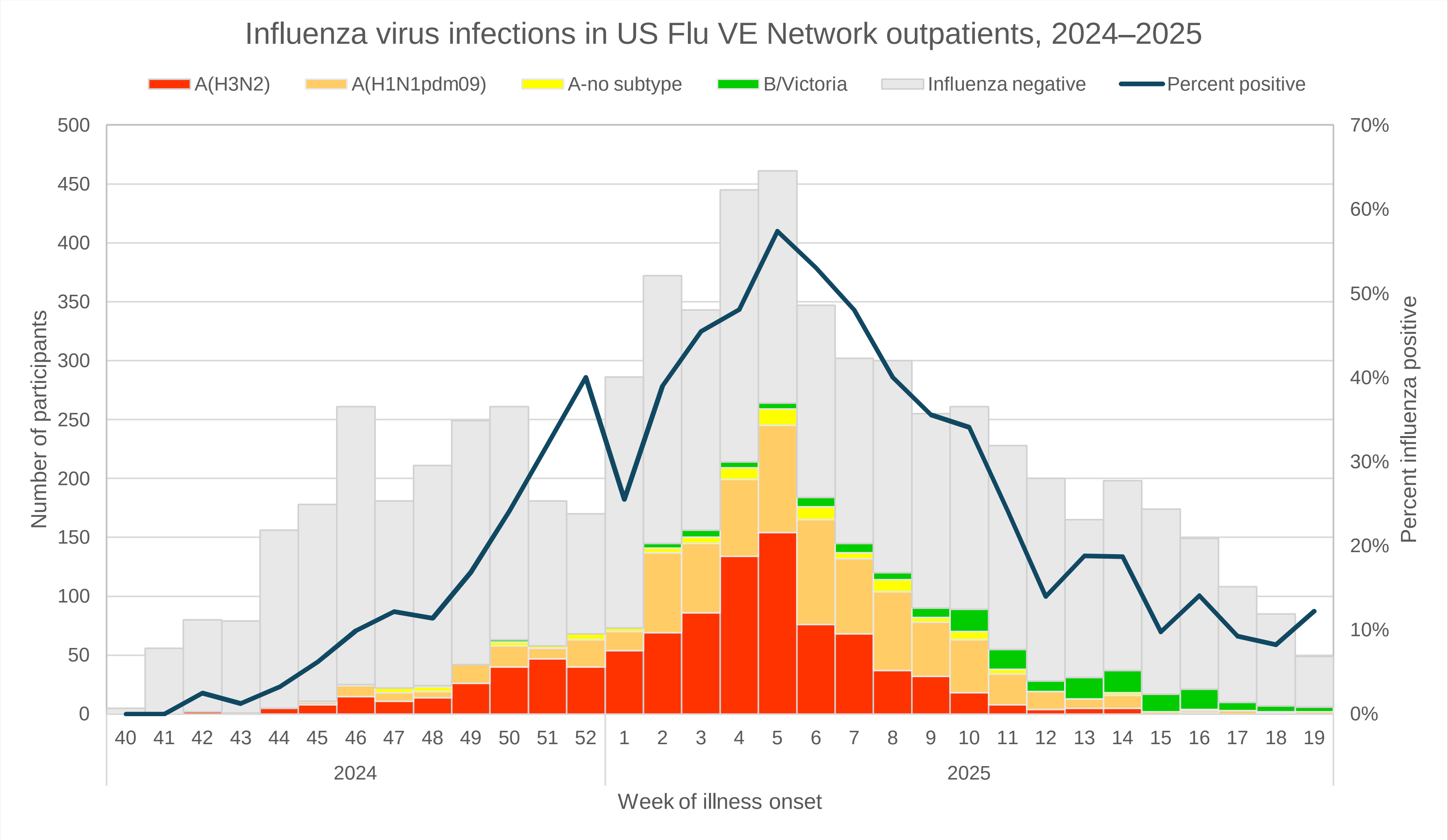

medRxiv preprint doi: https://doi.org/10.64898/2026.03.24.26348229; this version posted March 26, 2026. The copyright holder for this preprint (which was not certified by peer review) is the author/funder, who has granted medRxiv a license to display the preprint in perpetuity. This article is a US Government work. It is not subject to copyright under 17 USC 105 and is also made available for use under a CC0 license.

medRxiv preprint doi: https://doi.org/10.64898/2026.03.24.26348229; this version posted March 26, 2026. The copyright holder for this preprint (which was not certified by peer review) is the author/funder, who has granted medRxiv a license to display the preprint in perpetuity.
This article is a US Government work. It is not subject to copyright under 17 USC 105 and is also made available for use under a CC0 license.

Supplemental Table 3. Whole-genome sequencing results, US Flu VE Network, October 2024 – May 2025.

**Influenza Virus Subtype or Lineage**	**Genetic Characterization**				
	**Total No. of Subtype/Lineage Tested**	**HA Clade**	**Number (% of subtype/lineage tested)**	**HA Subclade**	**Number (% of clade tested)**
**A(H1N1)pdm09**	571	5a.2a	165 (29%)	C.1.9	53 (32%
				C.1.9.1	14 (8%)
				C.1.9.2	5 (3%)
				C.1.9.3	93 (56%)
		5a.2a.1	406 (71%)	D	8 (2%)
				D.1	1 (0.2%)
				D.3	359 (88%)
				D.5	39 (10%)
**A(H3N2)**	692	2a.3a.1	692 (100%)	J.1.1	1 (0.1%)
				J.2	479 (69%)
				J.2.1	6 (1%)
				J.2.2	111 (16%)
				J.2.3	10 (1%)
				J.2.4	8 (1%)
				J.2.5	77 (11%)
**B/Victoria**	117	3a.2	117 (100%)	C.3.1	50 (43%)
				C.3.2	2 (2%)
				C.5	4 (3%)
				C.5.1	32 (27%)
				C.5.6	13 (11%)
				C.5.6.1	5 (4%)
				C.5.7	11 (9%)

Supplemental Table 4. Influenza vaccine effectiveness against any outpatient influenza-associated illness visits among patients aged ≥8 months enrolled at US Influenza Vaccine Effectiveness Network sites, October 2024 through May 2025 by time since vaccination.

**Age group**	**Influenza Positive (Cases)**		**Influenza Negative (Controls)**		**Vaccine Effectiveness[a]**	
Time since vaccination (days)	**Total**	**No. Vaccinated (%)**	**Total**	**No. Vaccinated (%)**	**VE %**	**(95% CI)**
**All ages ≥8 months**						
14–59	61/1466	4	358/3403	11	56	(40 to 67)
60–120	200/1605	12	524/3569	15	41	(28 to 51)
>120	216/1621	13	580/3625	16	16	(-3 to 31)
**8 months –17 years**						
14–59	26/571	5	128/1367	9	55	(28 to 71)
60–120	58/603	10	172/1411	12	48	(28 to 63)
>120	56/601	9	160/1399	11	19	(-16 to 44)
**18–64 years**						
14–59	28/811	3	155/1737	9	56	(32 to 72)
60–120	99/882	11	233/1815	13	42	(24 to 56)
>120	95/878	11	249/1831	14	27	(2 to 45)
**≥65 years**						
14–59	7/84	8	75/299	25	47	(-32 to 79)
60–120	43/120	36	119/343	35	5	(-59 to 43)
>120	65/142	46	171/395	43	-35	(-131 to 21)

Abbreviations: CI, confidence interval; VE, vaccine effectiveness

[a] Models adjusted for study site, age, presence of ≥1 underlying health condition, and month of illness onset. 95% confidence intervals that exclude 0% are considered statistically significant.

medRxiv preprint doi: https://doi.org/10.64898/2026.03.24.26348229; this version posted March 26, 2026. The copyright holder for this preprint (which was not certified by peer review) is the author/funder, who has granted medRxiv a license to display the preprint in perpetuity. This article is a US Government work. It is not subject to copyright under 17 USC 105 and is also made available for use under a CC0 license.

Supplemental Table 5. Microneutralization titers to A(H3N2) virus A/Wisconsin/154/2024 and 95% confidence intervals among laboratory-confirmed A(H3N2)-positive participants compared to test-negative participants aged ≥18 years by current and prior season influenza vaccination status

	**Influenza-Positive (Cases)**		**Influenza-Negative (Controls)**	
	**N Tested**	**GMT (95% CI)**	**N Tested**	**GMT (95% CI)**
**All participants**	102	10 (8, 12)	130	19 (15, 24)
**Current season (2024–25) vaccination status**				
Vaccinated	36	17 (11, 27)	35	44 (28, 68)
Unvaccinated	66	7 (6, 8)	95	14 (11, 18)
**Current and prior season vaccination status**				
Vaccinated current 2024–25 only	20	18 (11, 30)	20	54 (29, 99)
Vaccinated current 2024–25 and prior 2023–24	16	17 (8, 37)	15	33 (17, 66)
Vaccinated prior 2023–24 only	4	6 (3, 10)	8	18 (5, 65)
Not vaccinated either 2024–2025 or 2023–24	62	7 (6, 8)	87	14 (10, 18)

Abbreviations: CI, confidence interval; GMT, geometric mean titer

medRxiv preprint doi: https://doi.org/10.64898/2026.03.24.26348229; this version posted March 26, 2026. The copyright holder for this preprint (which was not certified by peer review) is the author/funder, who has granted medRxiv a license to display the preprint in perpetuity. This article is a US Government work. It is not subject to copyright under 17 USC 105 and is also made available for use under a CC0 license.

Supplemental Table 6. Sensitivity analyses of influenza vaccine effectiveness against any influenza among participants of all ages

	Influenza Positive (Cases)		Influenza Negative (Controls)		Vaccine Effectiveness[a]	
	**Total**	**No. Vaccinated (%)**	**Total**	**No. Vaccinated (%)**	**VE %**	**(95% CI)**
**Primary analysis**	575/2016	29	1687/4777	35	33	(24 to 41)
**Varying exposure information source**						
Defining vaccination status using self-reported information only	450/1882	24	1239/4462	28	23	(11 to 33)
Defining vaccination status using documented information only	477/1964	24	1462/4707	31	37	(28 to 45)
**Varying participant inclusion criteria**						
Restricting to participants enrolled within 5 days of illness onset	480/1674	29	1176/3471	34	32	(21 to 41)
Restricting to participants enrolled with cough and fever	437/1680	26	801/2519	32	39	(28 to 47)
Include participants with known clinical results prior to enrollment	759/2432	31	1922/5409	36	28	(19 to 35)
Including SARS-CoV-2-positive participants	576/2035	28	1828/5143	36	35	(26 to 43)
Removing participants from Arizona	550/1720	32	1592/4178	38	33	(23 to 42)
Removing participants from Michigan	529/1900	28	1508/4437	34	34	(24 to 42)
Removing participants from Missouri	558/1952	29	1554/4282	36	34	(25 to 43)
Removing participants from Ohio	511/1664	31	1526/4009	38	35	(25 to 43)
Removing participants from Pennsylvania	373/1433	26	1287/3846	33	35	(24 to 44)
Removing participants from Texas	438/1610	27	1235/3641	34	37	(27 to 46)
Removing participants from Washington	491/1817	27	1420/4269	33	35	(25 to 43)

[a] Models adjusted for study site, age, presence of ≥1 underlying health condition, and month of illness onset. 95% confidence intervals that exclude 0% are considered statistically significant

medRxiv preprint doi: https://doi.org/10.64898/2026.03.24.26348229; this version posted March 26, 2026. The copyright holder for this preprint (which was not certified by peer review) is the author/funder, who has granted medRxiv a license to display the preprint in perpetuity. This article is a US Government work. It is not subject to copyright under 17 USC 105 and is also made available for use under a CC0 license.

medRxiv preprint doi: https://doi.org/10.64898/2026.03.24.26348229; this version posted March 26, 2026. The copyright holder for this preprint (which was not certified by peer review) is the author/funder, who has granted medRxiv a license to display the preprint in perpetuity.
This article is a US Government work. It is not subject to copyright under 17 USC 105 and is also made available for use under a CC0 license.

Supplemental Methods. Microneutralization assay

For MN assays, serial 2-fold dilutions from an initial 1:10 dilution (lowest detectable MN titer) were mixed with influenza viruses (100 50% tissue culture infective doses [TCID50]). The mixtures were infected with 1.5 x 104 Madin-Darby canine kidney-SIAT1 (MDCK-SIAT1) cells per well, and viral infection was determined by ELISA. Neutralizing antibody titers were defined as the reciprocal of the highest dilution of serum samples that achieved at least 50% neutralization. Samples with titer results <10 were assigned a titer of 5. MN assay viruses included the predominantly detected A(H3N2) virus among the US Flu VE Network sequenced viruses: MDCK-SIAT1 grown A/Wisconsin/154/2024.
